# Effect of chitosan on blood profile, inflammatory cytokines by activating TLR4/NF-κB signaling pathway in intestine of heat stressed mice

**DOI:** 10.1038/s41598-021-98931-8

**Published:** 2021-10-18

**Authors:** Sahar Ghulam Mohyuddin, Aftab Qamar, Can-ying Hu, Sheng-Wei Chen, Jia-ying Wen, Xiao-xi Liu, Xing-bin Ma, Zhi-chao Yu, Yan-hong Yong, Lian-Yun Wu, Ming-Long Bao, Xiang Hong Ju

**Affiliations:** 1 Department of Animal Science, College of Coastal Agricultural Sciences, Guangdong Ocean University, Zhanjiang, 524088 Guangdong China; 2 Shenzhen Institute of Guangdong Ocean University, Shenzhen, 518018 China; 3 Department of Veterinary Medicine, College of Coastal Agricultural Sciences, Guangdong Ocean University, Zhanjiang, 524088 Guangdong China

**Keywords:** Immunology, Diseases, Molecular medicine

## Abstract

Heat stress can significantly affect the immune function of the animal body. Heat stress stimulates oxidative stress in intestinal tissue and suppresses the immune responses of mice. The protecting effects of chitosan on heat stress induced colitis have not been reported. Therefore, the aim of this study was to investigate the protective effects of chitosan on immune function in heat stressed mice. Mice were exposed to heat stress (40 °C per day for 4 h) for 14 consecutive days. The mice (C57BL/6J), were randomly divided into three groups including: control group, heat stress, Chitosan group (LD: group 300 mg/kg/day, MD: 600 mg/kg/day, HD: 1000 mg/kg/day). The results showed that tissue histology was improved in chitosan groups than heat stress group. The current study showed that the mice with oral administration of chitosan groups had improved body performance as compared with the heat stress group. The results also showed that in chitosan treated groups the production of HSP70, TLR4, p65, TNF-α, and IL-10 was suppressed on day 1, 7, and 14 as compared to the heat stress group. In addition Claudin-2, and Occludin mRNA levels were upregulated in mice receiving chitosan on day 1, 7, and 14 of heat stress. Furthermore, the IL-6, IL-10, and TNF-α plasma levels were down-regulated on day 1, 7, and 14 of heat stress in mice receiving the oral administration of chitosan. In conclusion, the results showed that chitosan has an anti-inflammatory ability to tolerate hot environmental conditions.

## Introduction

Heat stress refers to the sum of a series of non-specific physiological responses exhibited by an animal to a heat source in an extremely high-temperature environment. The difference in stress intensity and stress time has a great impact on the health of the animal body. Mild and short-term heat stress significantly enhances animal resistance^1–3^. With the continuous warming of the global climate, combined with the increasing scale and stocking density of the breeding industry, the damage caused by heat stress to animals has become more serious and has become the most important stress-causing factor in southern China^4^. During heat stress in circulation, the number of heterophils and lymphocytes increases. Glucocorticoids facilitate the release of anti-inflammatory mediators, such as TNF-α, IL-10, and IL-4, and have apoptotic effects and strong anti-proliferative properties on immune cells^2^. Moreover, heat stress also affects the balance of anti-inflammatory and pro-inflammatory cytokines^3,5,6^. TLR4 mediated pathway plays a significant role in disease resistance but heat stress disturbs this pathway and has negative consequences on the performance of the animal. TLR activates MYD88 molecules that involve in the initiation of nuclear transcription factors (NF-κB) which induce the inflammatory mediators and suppress the immune responses^7^. TLR4 after LPS secretion in circulation initiates NF-κB to promote the overproduction of anti and pro-inflammatory cytokines. Therefore, it is essential to reduce inflammatory status by preventive the initiation of TLR4 mediated NF-κB.

Chitin is the main component of crustaceans such as shrimps and crabs. It is also commonly found in insect epidermis and fungal cell walls. It is the second-largest polymer in the world after cellulose^8^. Chitosan (COS) produced by deacetylation of chitin is the only cationic polysaccharide in nature with a linear structure. It has good biocompatibility and biodegradability, in the pharmaceutical industry, but its larger molecular weight, poor solubility, and higher viscosity limit its application in various fields^9^. Chitosan is a low-molecular substance with a degree of polymerization of 2–20, obtained by hydrolysis or enzymatic hydrolysis of chitosan, with an average molecular weight of less than 3900 Da^10^. Chitosan can also be used as a gene therapy carrier^11^ and has an important position in medical treatment. It is generally believed that COS can exert biological activity through two ways of absorption into the blood and the intestine^12^. After COS is absorbed into the blood in the small intestine, it reaches the target site and exerts anti-inflammatory, immune, antioxidant, antiviral, and anti-tumor effects through signaling pathways^13^. A study by Zacour et al. stated that oral administration of chitosan can decrease the body weight and fat mass inside intestinal tissue^14^. COS has certain advantages like decreasing intestinal swelling, improve cell-mediated immune response^15^, inhibition of edema by depletion of carrageenan induce inflammation^16^, and improvement of hypersensitive inflammation^17^. A Study described by Xie et al. showed that COS has anti-oxidative, antibacterial, anti-allergic, and anti-tumor properties^18^. In another study, pigs were administrated with COS and there was evidence of increased growth performance^19^. It can also significantly increase the expression level of intestinal tight junction proteins^20^, which has great development for the prevention and control of animal inflammatory bowel disease. However, there are few research reports on the chitosan effect, its effective dose, and its mechanism on inflammatory bowel disease. In this study, we reported the effects of COS on Heat stress induced inflammation in colonic tissue and immune pathways, including the serum inflammatory cytokines response. The protecting effects of COS on heat stress induced colitis have not been reported therefore the purpose of this study was to investigate the protective effects of COS on heat stress-induced colitis.

## Results

### Effect of chitosan on body weight and colon length

**Figure 1:**
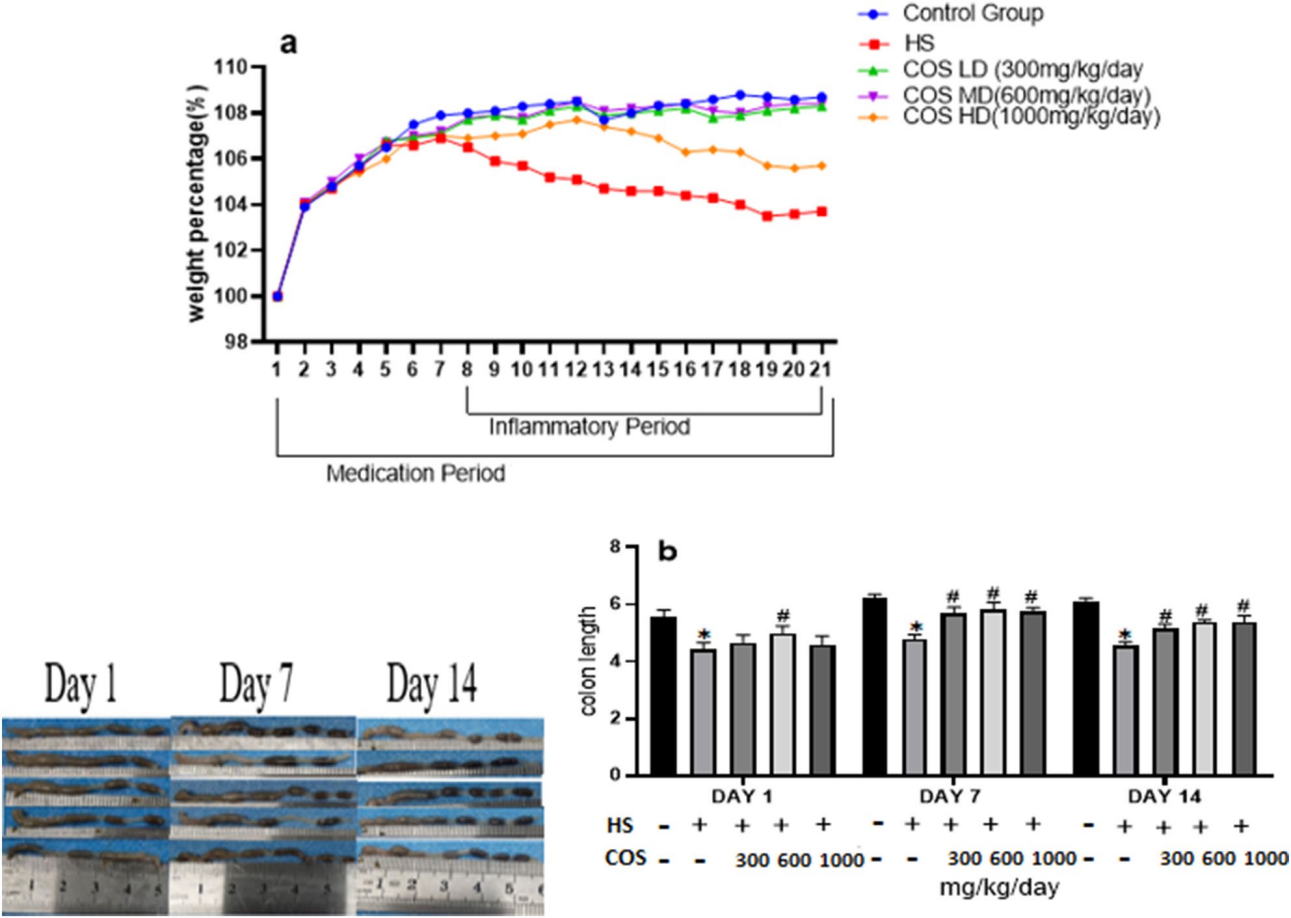
Effect of chitosan treatment on the mice. (**a**) Body weight (**b**) Colon length. Control: normal mice; HS: Heat stress mice; COS-LD: mice treated with HS plus COS (300 mg/kg); COS-MD: mice treated with HS plus COS (600 mg/kg); COS-HD: mice treated with HS plus COS (1000 mg/kg). Data are expressed as mean ± standard error. *Indicates *p* < 0.05 compared with the blank control group, and # indicates *p* < 0.05 compared with the HS control group.

After exposure to heat stress, the mice showed signs of depression, dry hair, reduced feed intake, increased water intake, lethargy, and weight loss. The present study showed that the weight of the mice in the control group increased steadily, and the body weight of the heat-stressed mice showed a downward trend during the whole exposure of heat stress. However, among all the chitosan treated groups LD and MD doses were suitable to increased body weight trend as compared with heat stress group. Whereas, there was no significant (*p* < 0.05) differences in body weight of mice in HD group as compared to the heat stress group due to higher concentration of chitosan (Fig. 1a). The results of the present study showed that chitosan has ability to improve body weight.

The colon length of the heat stress group was significantly reduced as compared with the control group (*p* < 0.05). However, the mice receiving oral administration of chitosan showed a significant increase in the colon length as compared to the heat stress group. Chitosan improved the effects of heat stress on colon length shortening (Fig. 1b).

### Effect of chitosan on organ index

**Figure 2:**
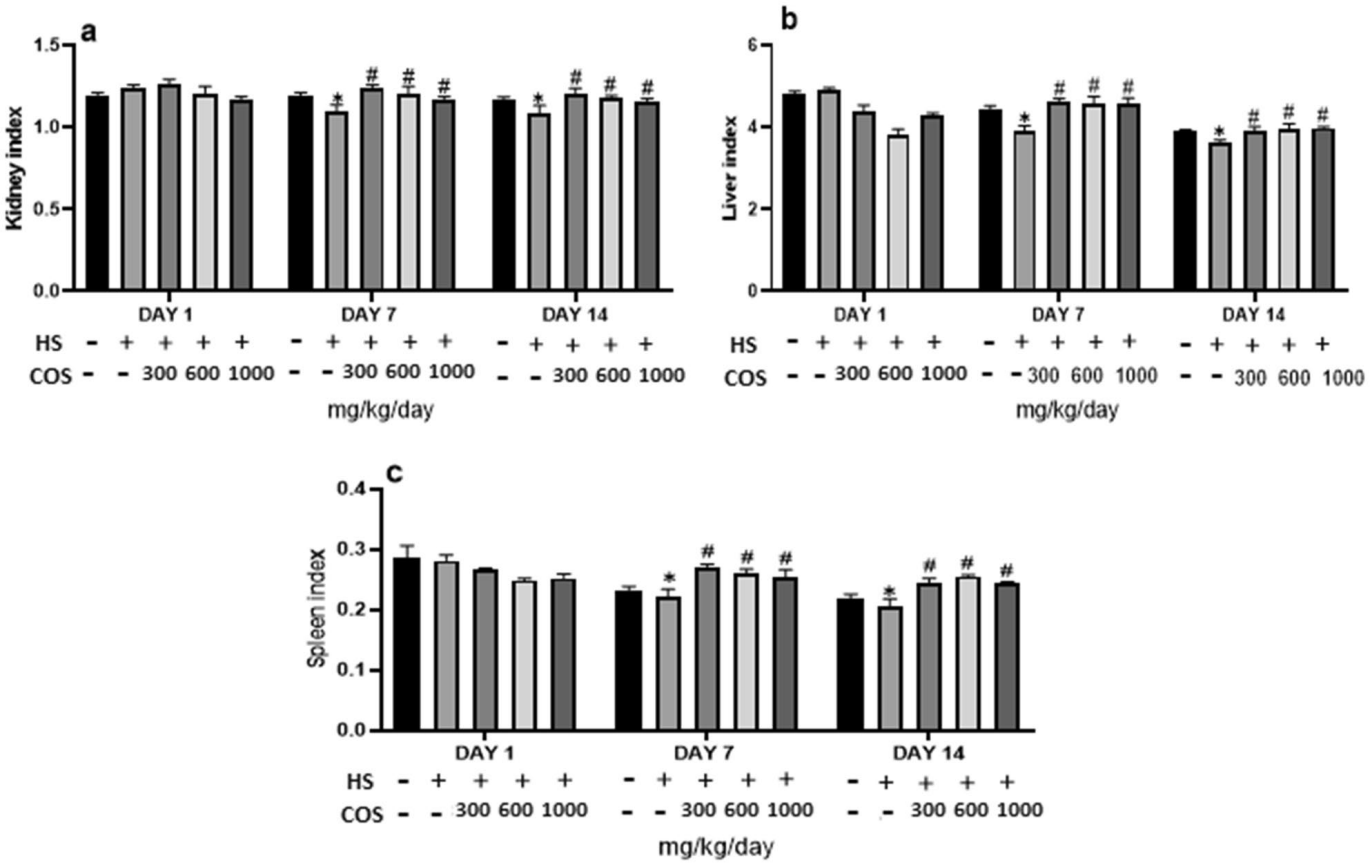
Effect of chitosan treatment on the organ index. Control: normal mice; HS: Heat stress mice; COS-LD: mice treated with HS plus COS (300 mg/kg); COS-MD: mice treated with HS plus COS (600 mg/kg); COS-HD: mice treated with HS plus COS (1000 mg/kg). Data are expressed as mean ± standard error. *Indicates *p* < 0.05 compared with the blank control group, # indicates *p* < 0.05 compared with the HS control group.

Moreover, the result of the present study also showed that heat stress group had decreased kidney weight, on day 7 and 14 of the heat stress period as compared to the control group. But in all chitosan treated groups had significantly (*p* < 0.05) improved kidney weight on day 7 and 14 of the experiment when compared with heat stress group (Fig. 2a).

Furthermore, the result of the present study also showed that heat stress group had decreased liver weight, on day 7 and 14 of the heat stress than control group. But all the chitosan treated groups showed a significant increase (*p* < 0.05) in the liver weight on day 7 and 14 of experiments when compared with the heat stress group and control group (Fig. 2b).

Moreover, the result of the present study also showed that the heat stress group had decreased spleen weight, on day 7 and 14 of the heat stress period as compared to the control group. But in all chitosan treated groups had improved spleen weight on day 7 and 14 of the experiment when compared with heat stress group. Though when related chitosan group with the control group, all treatment groups of chitosan had significantly (*p* < 0.05) enhanced spleen weight than the control group. However, there was no significant difference in organ weight on day 1 as compared to heat stress group. The results of the present study showed that chitosan can recover the weight of the organs and provide safety to the body against the production of reactive oxygen species under heat stress. The effect of chitosan on organs is shown in Fig. 2c.

### Effect of chitosan on colonic tissue histology of heat stressed mice

**Figure 3:**
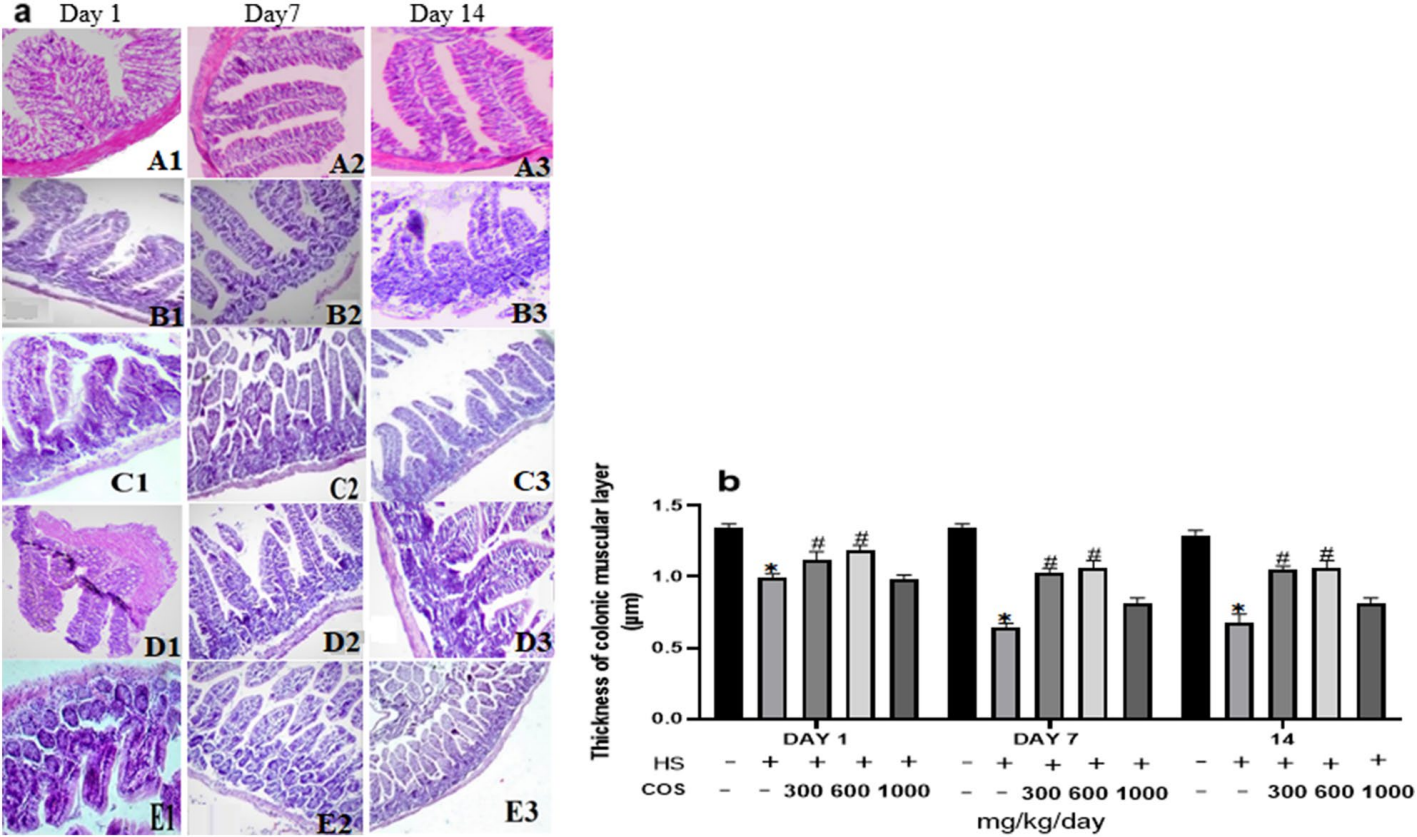
Chitosan attenuates heat stress-induced colitis mice (HE, 100 ×). (**A**) control group (**B**) Heat Stress (**C**) COS LD + HS (**D**) COS MD + HS (**E**) COS HD + HS, 1: 1st day, 2: 7th day, 3: 14th day. Data are expressed as mean ± standard error. *Indicates *p* < 0.05 compared with the blank control group, ^#^Indicates *p* < 0.05 compared with the HS control group.

The thickness of the mucus layer of the colon in the heat stress group was significantly reduced as compared to the control group, while that of the chitosan groups the thickness was increased on day 1, 7, and 14 than heat stress group (Fig. 3). Histopathological examination found that compared with the control group, the muscle layer thickness, villi height, and the number of goblet cells were significantly reduced in the heat stress group, while these changes in chitosan LD and MD groups were increased than those in the heat stress group. Among all the chitosan treated groups LD and MD doses were suitable to improve the intestine histology. Whereas, there were no significant (*p* < 0.05) differences in colonic tissue structure in HD as compared to heat stress group due to a higher concentration of chitosan.

### Effect of chitosan on white blood cell

**Figure 4:**
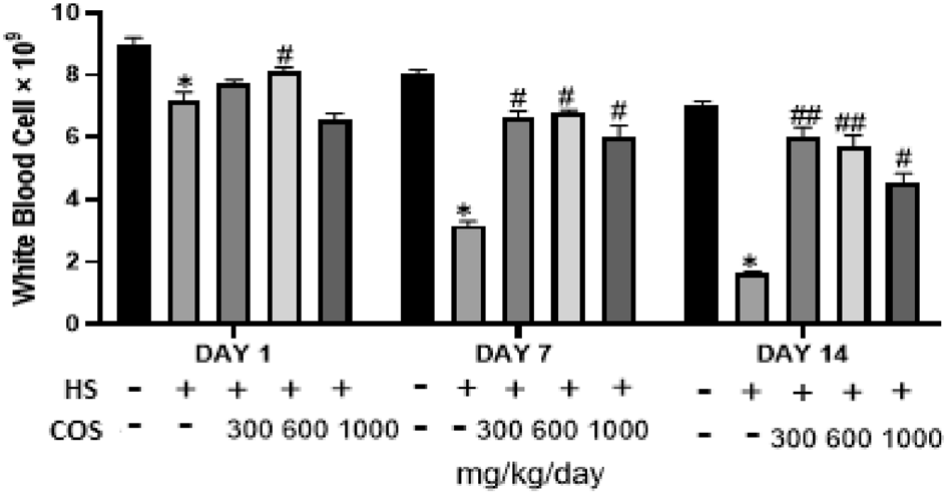
Effect of chitosan treatment on WBC. Control: normal mice; HS: Heat stress mice; COS-LD: mice treated with HS plus COS (300 mg/kg); COS-MD: mice treated with HS plus COS (600 mg/kg); COS-HD: mice treated with HS plus COS (1000 mg/kg). Data are expressed as mean ± standard error. *Indicates *p* < 0.05 compared with the blank control group, ^##^Indicates p < 0.01 and ^#^Indicates p < 0.05 compared with the HS control group.

The results showed that the number of WBC was significantly (*p* < 0.05) decreased in heat stress group as compared to the control group. On the other side, the WBC number was significantly increased in all treated chitosan groups on day 7, and 14 of the experiment when compared with the heat stress group. However, there was no difference in WBC number in HD as compared to heat stress group on day 1 but on day 7 and 14 there was a significant difference in WBC number. In conclusion, all chitosan groups were suitable to increase WBC values. However, the results of the present study showed that chitosan has antioxidants capability which protects tissues from oxidative damages and improve immunity which is reflected by increased WBC number in heat stressed chitosan treated mice. The effect of chitosan on WBC is shown in Fig. 4.

### Effect of chitosan on lymphocytes

**Figure 5:**
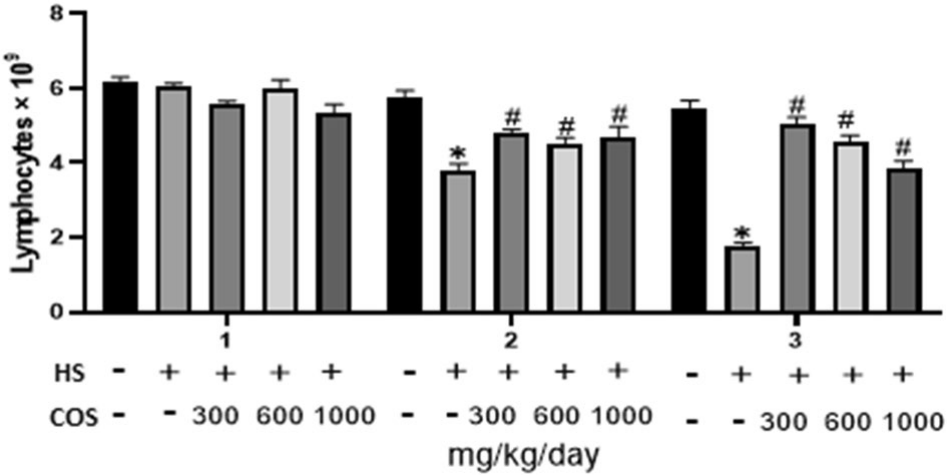
Effect of chitosan treatment on lymphocytes. Control: normal mice; HS: Heat stress mice; COS-LD: mice treated with HS plus COS (300 mg/kg); COS-MD: mice treated with HS plus COS (600 mg/kg); COS-HD: mice treated with HS plus COS (1000 mg/kg). Data are expressed as mean ± standard error. *Indicates *p* < 0.05 compared with the blank control group, ^#^Indicates* p* < 0.05 compared with the HS control group.

Lymphocytes in blood were significantly (*p* < 0.05) decreased in the heat stress group as compared to the control group. While, lymphocytes number was significantly (*p* < 0.05) increased in all chitosan treated groups on day 7, and 14 of the experiment when compared with heat stress group. However, there was no difference in lymphocyte number as compared to the heat stress group on day 1, but on day 7, and 14 there was a significant difference in lymphocyte number. In conclusion, all chitosan groups were suitable to increase lymphocyte number (Fig. 5).

### Effect of chitosan on serum cytokines (TNF-α, IL-10, IL-6) analysis

**Figure 6:**
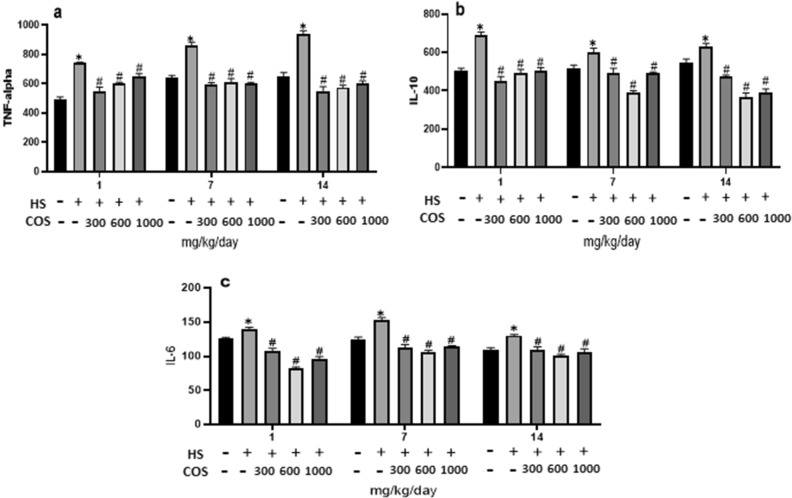
Effects of chitosan on the inflammatory cytokines. (**a**) TNF-alpha (**b**) IL-10 (**c**) IL-6. Control: normal mice; HS: Heat stress mice; COS-LD: mice treated with HS plus COS (300 mg/kg); COS-MD: mice treated with HS plus COS (600 mg/kg); COS-HD: mice treated with HS plus COS (1000 mg/kg). Data are expressed as mean ± standard error. *Indicates* p* < 0.05 compared with the blank control group, ^#^Indicates *p* < 0.05 compared with the HS control group.

Serum inflammatory cytokines (IL-10, IL-6, and TNF-α) response in mice was increased in the heat stress group as compared to the control group. Whereas, heat stressed mice with oral administration of chitosan had significantly (*p* < 0.05) decreased inflammatory cytokines response (IL-10, IL-6, TNF-α when compared with heat stress group on day 1, 7, and 14 of experiment. The results showed that chitosan has an excellent anti-inflammatory capability which inhibits the level of inflammatory cytokines induced by stressful conditions. The effect of chitosan on inflammatory cytokines is shown in Fig. 6.

### Effect of chitosan on expressions of HSP70

**Figure 7:**
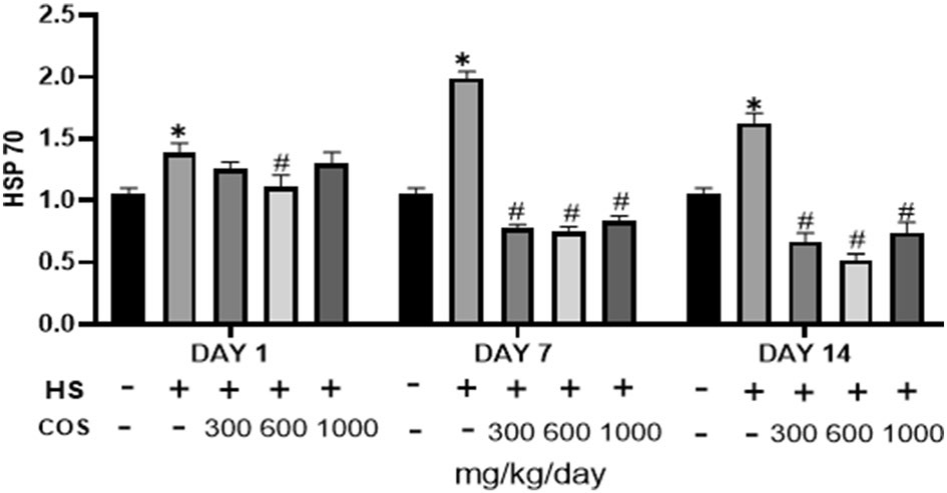
Effects of chitosan on protein expression of HSP70. Control: normal mice; HS: Heat stress mice; COS-LD: mice treated with HS plus COS (300 mg/kg); COS-MD: mice treated with HS plus COS (600 mg/kg); COS-HD: mice treated with HS plus COS (1000 mg/kg). Data are expressed as mean ± standard error. *Indicates *p* < 0.05 compared with the blank control group, ^#^Indicates *p* < 0.05 compared with the HS control group.

The results showed that the heat stress group had significantly increased the mRNA level of HSP70 in colonic tissue in mice as compared to the control group but the results of the current study explored that all the chitosan treatment groups had significantly reduced mRNA expression of HSP70 as compare to the heat stress group Fig. 7.

### Chitosan prevent HS mice from inflammatory response

**Figure 8:**
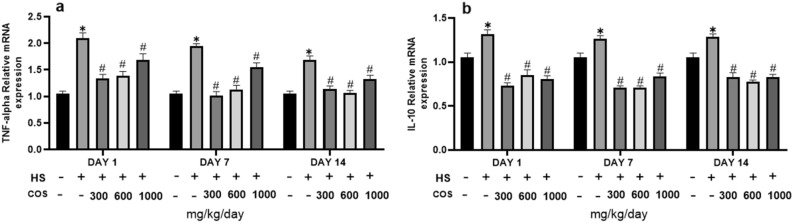
Effects of chitosan on protein expression of IL-10 and TNF-α. Control: normal mice; HS: Heat stress mice; COS-LD: mice treated with HS plus COS (300 mg/kg); COS-MD: mice treated with HS plus COS (600 mg/kg); COS-HD: mice treated with HS plus COS (1000 mg/kg). Data are expressed as mean ± standard error. *Indicates *p* < 0.05 compared with the blank control group, ^#^Indicates *p* < 0.05 compared with the HS control group.

The results showed that the heat stress group showed a significant increase in the expression of TNF-α and IL-10 in colonic tissues as compared with the control group. On the other hand, as compared with the heat stress group, all treatment groups had significantly lower expression of inflammatory cytokines (Fig. 8a,b). Treatment with chitosan significantly suppressed the level of IL-10 and TNF-α. These findings indicated that chitosan exerted preventive action against the inflammatory responses in intestinal mucosa via inhibition of NF-κB activation.

### Effect of chitosan on tight junction protein

**Figure 9:**
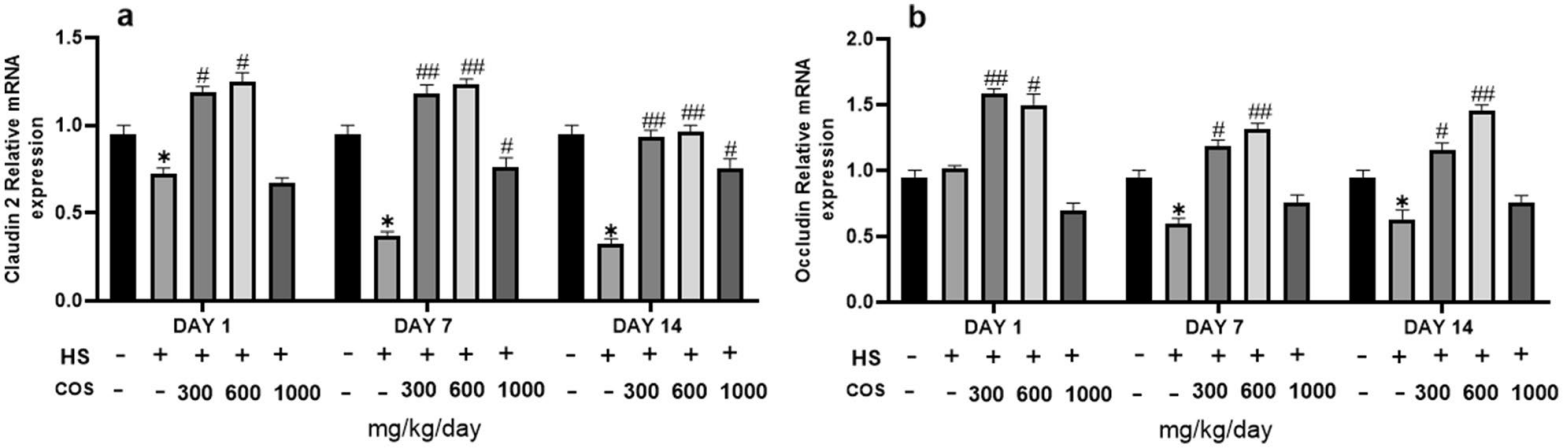
Effects of chitosan on protein expression of claudin-2 and occluding. Control: normal mice; HS: Heat stress mice; COS-LD: mice treated with HS plus COS (300 mg/kg); COS-MD: mice treated with HS plus COS (600 mg/kg); COS-HD: mice treated with HS plus COS (1000 mg/kg). Data are expressed as mean ± standard error. *Indicates *p* < 0.05 compared with the blank control group, ^#^Indicates *p* < 0.05 and ^##^Indicates p < 0.01 compared with the HS control group.

The results showed that the heat stress group significantly down-regulated mRNA expression of Claudin-2 in colonic tissue on day 1, 7, and 14 as compared to the control group. While mRNA expression of Occludin there was no changes in heat stress group on day 1 but on day 7, and 14 the heat stress group showed a significant decrease in the expression of Occludin than the control group. On the other hand, as compared with the heat stress group, all chitosan treatment groups had significantly upregulated the expression of Claudin-2 in colonic tissue but the expression of Occludin in chitosan LD and MD groups was increased on day 1, 7, and 14 than control group (Fig. 9). These results showed that chitosan was suitable to reduce the inflammation in colonic tissue. These findings indicated that chitosan has the ability to reduce the harmful effects of heat stress and preventive action against the inflammatory responses in intestinal mucosa via upregulated the tight junction protein.

### Effect of chitosan on expressions of TLR4 and p-65

**Figure 10:**
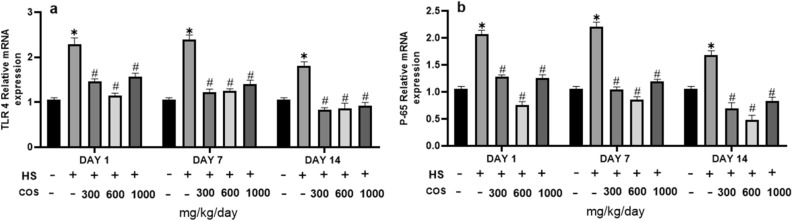
Effects of chitosan on protein expression. (**a**) TLR4 (**b**) NF-κB**.** Control: normal mice; HS: Heat stress mice; COS-L: mice treated with HS plus COS (300 mg/kg); COS-M: mice treated with HS plus COS (600 mg/kg); COS-H: mice treated with HS plus COS (1000 mg/kg). Data are expressed as mean ± standard error. *Indicates *p *< 0.05 compared with the blank control group, ^#^Indicates *p* < 0.05 compared with the HS control group.

The results showed that the heat stress group had significantly increased the mRNA level of TLR4 as compared to the control group but the results of the current study explored that in all chitosan treatment groups the mRNA expression of TLR4 was significantly reduced than heat stress (Fig. 10a).

The effect of COS on the nuclear translocation of NF-κB (p65) under heat stress showed that as compared to the control group, heat stress group had significantly increased the mRNA level of p65, while the mRNA expression of p65 in all chitosan treatment groups significantly reduced (*p* < 0.05) (Fig. 10b).

## Discussion

Heat stress is an alarming situation that could lead to the death of a living organism^21^. Moreover, the unfavorable influence of heat stress will get worse with the rising issue of global warming^22^. Heat stress leads to oxidative stress characterized by excessive reactive oxygen species production and the impaired antioxidant capacity^23,24^. For broilers, heat stress shows a decrease in feed intake, feed conversion rate, and growth rate^25^, while in laying hens, there is a decrease in egg production rate, and egg weight. In the pig industry, heat stress causes damage to the reproductive performance of boars, which is manifested in decreased semen quality, sperm density, and sperm motility, and increased sperm deformity rate. It can be seen that heat stress has a serious impact on animal production performance. Therefore, it is of great significance to analyze the mechanism of heat stress and develop targeted prevention and control technology.

At present, there are few studies on the protective effect of COS on the intestinal, and the mechanism of action is not clear. Therefore, in this study, mouse intestinal tissue was used as a model for *in-vivo* experiment, and heat stress induction was used to establish an inflammation model. Administration of chitosan at different concentrations was used to explore the effect of chitosan on mouse colitis. The current study showed that measurement of the colon length and body weight was decreased in the heat stress group as compared with the control group. However, in mice receiving chitosan, the colon length was significantly higher as compared to the heat stress group. Chitosan improved the effects of heat stress on colon length shortening and the body weight of mice. This study's results were similar to Lin Shi et al. who described that COS positively affects the colon length and body weight of mice under DSS-induced colitis^26^. A study showed that oral administration of chitosan also enhances the body weight in rats^27^ and mice ^28^.

In our study, the results showed that the number of WBC was significantly (*p* < 0.05) decreased in the heat stress group while WBC number was significantly (*p* < 0.05) increased in all chitosan treatment groups on day 7, and 14 of the experiment when compared with the heat stress group. the Our study results were similar to the study of Yeh et al. which described that oral administration of chitosan can increase the number of white blood cells in mice^29^.

Lymphocytes play an important role in various immunological functions like the production of antibodies and the regulation of defense mechanisms. Heat stress could negatively affect the proliferation of lymphocytes^30,31^. A study showed that there was an increase in the number of lymphocyte and neutrophil count in Sahiwal and Karan Fries heifers when exposed to 40 °C^32^. Our study results were similar to these findings, while the number of lymphocytes was significantly increased in all COS groups as compared to the heat stress group. In a study described by Rana et al. under heat stress there was no significant difference in white blood cell in sheep^33^, while in our study the number of lymphocytes and white blood cells were significantly decreased in heat stress group. A study by Elvinger et al. stated that the number of lymphocytes were significantly reduced in Holstein cows after 60 h exposure of heat stress (42 °C)^34^. Moreover, Lacetera et al. described that at 41–42 °C lymphocyte production under *in-vitro* conditions decreased in both Brown Swiss and Holstein cows^35^. Our study results were similar to these finding. Serum inflammatory cytokines (IL-10, IL-6, and TNF-alpha) response in mice was increased in heat stress group as compared to the control group. Whereas, heat stressed mice with oral administration of chitosan had significantly (*p* < 0.05) decreased inflammatory cytokines response (IL-10, IL-6, TNF-alpha) when compared with heat stress group on 1, 7, and 14 day of experiment. Our study results were similar to Liu et al. who described that COS successfully reduced acute colitis by suppressing the high production of TNF-α, IL-6, and IL-10 in colon tissue in mice^36^. A study showed that heat-stressed rats have an elevated level of pro-inflammatory cytokines like IL-1β, IL-6, and TNF-α in the gut^37,38^. In addition, heat stress has been reported to induce increased blood cortisol concentrations which have been shown to inhibit the production of cytokines such as IL-4, IL-5, IL-6, IL-12, IFN-γ, and TNF-α in dairy cows^39^. These results are similar to our study results. The level of anti-inflammatory cytokines IL-10 was significantly reduced in heat-stressed rats. Thompson et al., (2014) described that increased expression of IL-10 in heat-stressed cows related to cows housed under thermo-neutral conditions^40^, while in our study the expression of IL-10 was significantly increased in heat-stressed mice.

Heat shock factors turn on a cascade of responses like regulating the expression of heat shock protein^41^. HSP70 plays a main role in the adaptive response to heat stress by improving the antioxidant ability, inhibiting lipid peroxidation, and upregulated the activity of digestive enzyme action^42^. In a previous study, the mRNA expression of HSP70 was significantly higher in the intestine of heat stressed mice^43^. A study described by Varasteh showed that there was no heat stress-related changes in HSP70 and HSP90 mRNA expression in the duodenum and colon of chicken^44^. However, our study results showed that the heat stress group had significantly increased the mRNA level of HSP70 in colonic tissue in mice as compared to the control group but the results of the current study explored that all chitosan treatment groups significantly reduced mRNA expression of HSP70 as compared to the heat stress.

A study described by Wan et al. that COS can prevent the inflammatory response of IL-6 and TNF-α in the mucosa of jejunum and ileum resulting from *Enterotoxigenic Escherichia coli* infection in weaned pigs^45^. In another study described by Lin shi et al. inflammatory cytokines, such as IL-6 and IL-8, were increased in IPEC-J2 cells after LPS exposure. And a low dose of chitosan (200 μg/mL) was effective to decrease the cytokines response^26^. In our study chitosan suppressed the expression of TNF-α and IL-10 in heat-stressed mice. Moreover, a study by Yu et al., (2013) stated that under heat stress, the gene expression of IFN-γ, IL-10, and TNF-α was significantly elevated in rat liver^46^, which showed that inflammatory cytokines production is stimulated by heat stress.

TLR4, a stress-related biosensor in initial injury response^47^, plays a key role in stimulating inflammatory cytokines production^48^. It is reported that HSP70 activates the NF-κB but with the addition of TLR4 blockers^42^. TLR4 plays an important role in immunity, but the role of TLR4 in the pathogenesis of heat stress is not clear. In a previous study, the mRNA expression of TLR4 and plasma level of inflammatory cytokines increased in heat-stressed pigs^49^. These results were similar to our study results. In our study, the mRNA expression of TLR4 increased in heat-stressed mice. In a previous study, the expression of NF-κB and TNF-α increased in the mouse hippocampus under heat stress^50^. While in our study the expression of p65 and TNF-α also increased in colonic tissue of mice under heat stress and chitosan have potential to inhibit these effect. In another study the mRNA expression of TLR2, TLR4 was significantly reduced in heat-stressed rat^3^. While in our study the mRNA expression of TLR4 was significantly increased in heat-stressed mice. Several studies were conducted on the role of chitosan to reduce LPS-induced colitis in mice^51^. The NF-κB signaling pathway has a vital role in the regulation of inflammatory functions^52^. For the first time, this study evaluates the effects of chitosan against heat stress. COS can inhibit the expression of inflammatory genes by inhibiting NF-κB induced by lipopolysaccharide (LPS). Yang et al. found that COS can counteract the oxidative damage, inflammation, and apoptosis of Caco-2 cells induced by LPS^53^. COS can improve the colon shortening and tissue damage in mice induced by LPS and it can also inhibit the activation of NF-κB, expression of TLR4, and inflammatory cytokines^26^. The results showed that the heat stress group had significantly increased the mRNA level of TLR4, p65, as compared to the control group but the results of the current study explored that chitosan treatment groups had significantly reduced mRNA expression of TLR4. The effect of COS on the nuclear translocation of NF-κB (p65) under heat stress showed that as compared to the control group, the heat stress group had significantly increased the mRNA level of p65, while the mRNA expression of p65 in chitosan treatment groups significantly (*p* < 0.05) reduced.

Tight Junction (TJ) that may destroy the epithelial structure through cytotoxicity changes the position of TJ in the colonic mucosa to allow the flora to penetrate and induce inflammation. In addition, the anti and pro-inflammatory factors IL-6 and TNF-alpha can also significantly reduced the expression of TJ, such as Claudin-2 and Occludin weakens the mucosal barrier, allowing bacteria to enter the colon tissue and cause inflammation^54^. Studies have been shown that tight junction protein expression is closely associated with the TLR4 signaling pathway^3^, and can down-regulate the expression of tight junction proteins. The results showed that the heat stress group had significantly down-regulated the expression of claudin-2, and occludin protein in colonic tissue on day 1, 7, and 14 as compared with the control group. On the other hand, as compared to the heat stress group, LD and MD groups had significantly upregulated expression of tight junction protein in colonic tissue. But, there was no difference in HD dose as compared to heat stress group due to high concentration. These findings indicated that LD and MD groups has the ability to reduce the harmful effects of heat stress and preventive action against the inflammatory responses in intestinal mucosa via upregulated the tight junction protein.

In conclusion, our study results showed that heat stressed mice with oral administration of chitosan had increased heat tolerance and immunity as compared to heat stress which is revealed by improved colonic histology, and suppression of serum inflammatory cytokines response, and increased organ weight, blood parameters, reduced mRNA expression of TLR4 and its downstream gene expression as well as decreased p65, IL-10, TNF-α, and increased the Occludin, and Claudin-2 expression. It is concluded that chitosan could protect mice against HS-induced colitis. Chitosan significantly inhibited heat stress-induced inflammatory cytokines production. The mechanism was related to the activation of TLR4, which lead to the inhibition of the NF-κB signaling pathway.

## Materials and methods

### Chemicals and reagents

CHITOSAN (average molecular weight < 1000, > 90% degree of deacetylation) was purchase by Zhong Tai He Technology (Beijing, China). Mouse TNF-α and IL-10, IL-6 ELISA kit were purchased from Shanghai Enzyme-Linked Biological Company. TRIZOL was purchased from Thermo Fisher; PRIMESCRIPT RT REAGENT KIT with gDNA ERASER. TOP GREEN qPCR SuperMix was purchase from TranStart (transgen.com.cn). In addition, a REVERSE TRANSCRIPTION cDNA KIT was also purchased from TranStart.

### Experimental groups

Sixty SPF male mice (C57BL/6 J), were kept in an environmentally controlled room. Mice were divided into three groups; thermo-neutral control (CG) group, high temperature (HS) group, heat stress and chitosan group (LD: 300 mg/ kg/day, MD: 600 mg/kg/day, HD: 1000 mg/kg/day). Each group has two replicates having 6 mice/replicate. The mice in thermo-neutral (CG) group were kept at comfort temperatures (24 ± 1 °C) and relative humidity (65–85%). After one week of adaptation, the experimental animals were exposed to chitosan for 7 days without heat stress. The mice in the heat stress and chitosan treatment group were kept at high temperature (40 ± 1 °C for 4 h/day) and relative humidity (65–85%) for 14 consecutive days. The body weight of mice from each group was recorded on daily basis. Mice were sacrificed on day 1, 7, and 14 of heat stress. Mice were reared in cages and shifted in a disinfected well-ventilated room. Electric heaters were arranged in the experimental mice room to regulate the environmental temperature. All experimental protocols were approved by the Animal Ethics Committee of Guangdong Ocean University, China, and were performed according to the ethical guidelines of the European Community guidelines.

### Assessment of colitis

During heat stress exposure, body weight was measured daily. At the end of the experiment, mice were sacrificed by cervical dislocation, blood sample and colon of each mouse were collected. The length of the colon was measured and then washed instantly using physiological saline. 5 mm length of the colon tissue were cut and fixed in 10% formalin solution. A part of colon tissue was stored at − 80 °C for mRNA expression. After fixing in formalin solution for 24 h, colon tissue was dehydrated in different series of alcohol solutions and then embedded in paraffin wax for histopathological analysis, after that the tissue was sliced up to 4 μm by using a microtome and mounted on the slide then stained with Hematoxylin and Eosin (H&E). The stained slices were covered with coverslips using neutral balsam as an adhesive. Stained colon tissue slides were examined under a light microscope to check the severity of intestinal tissue inflammation, the degree of mucosal injury, and crypt damage. Mucus and muscle layer thickness was measured by Image-Pro Plus 6.0 software (Media Cybernetics, Inc., Rockville, MD, USA).

### Organ weight

The desired organs were collected by dissecting the mice body and organs weight was recorded immediately in grams on day 1, 7, and 14 of the experiment.

$${\text{Intestine }}\;{\text{index }} = \, \left( {{\text{Intestine }}\;{\text{weight}}/{\text{body }}\;{\text{weight}}} \right) \, \times { 1}00\%$$

$${\text{Liver }}\;{\text{index }} = \, \left( {{\text{Liver}}\;{\text{ weight}}/{\text{body }}\;{\text{weight}}} \right) \, \times { 1}00\%$$

$${\text{Spleen }}\;{\text{index }} = \, \left( {{\text{Spleen }}\;{\text{weight}}/{\text{body }}\;{\text{weight}}} \right) \, \times { 1}00\%$$

### Lymphocyte and white blood cell count

Four mice from each of the experimental groups were selected every time for obtaining the blood sample. About 25 μL of blood was collected from the tail vein with a micro-sampler and the number of lymphocytes and WBCs was analyzed by using an automatic blood cells analyzer (URIT-5180, Medical Electronic Co., Ltd., Guilin, China).

### ELISA assay

The levels of inflammatory cytokines TNF-α, IL-6, and IL-10 were detected by the ELISA kit according to the instructions of the manufacturer Shanghai Enzyme-Linked Biological Company.

### Quantitative PCR

Total RNA was isolated from the colonic tissue using TRIZOL reagent with iPrep instrument (Takara, Dalian, China). The quality of the isolated RNA was determined by spectrometry (A260/A280) using an ND-1000 spectrophotometer (NanoDrop Technologies, Wilmington, DE, USA) and electrophoresis of RNA. Isolated total RNA was reverse transcribed to cDNA using an EASYSCRIPT ONE-STEP gDNA REMOVAL and cDNA SYNTHESIS SUPERMIX KIT (Takara, Dalian, China). The resulting cDNA was subjected to qPCR and the sequence of primer (Sangon Biotech CO., Ltd., Shanghai, China) used are listed in Table 1. Reactions were performed on a Bio-Rad CFX connect real-time PCR detection system (Bio-Rad Hercules, CA, USA). The qPCR amplification conditions were as follows: pre-denaturation at 94 °C for 30 s, followed by 45 cycles of denaturation at 94 °C for 5 s, annealing at 55 °C for 15 s, and elongation at 72 °C for 10 s. Expression levels of IL-10, TNF-α, TLR-4, P-65 were calculated by the 2^−ΔΔCT^ method.

**Table 1:** Sequence of primer used for detection of mRNA.

Primer name	Forward (5′–3′)	Reverse (5′–3′)
TNF-α	GGACTAGCCAGGAGGGAGAACAG	GCCAGTGAGTGAAAGGGACAGAAC
IL-10	TTCGCTGATGATGCTTAGTTT	ACAGGCAATGACGAAATA
TLR4	CACAGAAGAGGCAAGGCGACAG	GAATGACCCTGACTGGCACTAACC
P-65	GTCCCCGTGCCCTCTGTCTAAG	ACTGTTCCTGGTCCTGTGTAGCC
HSP 70	GTGGTGAACGACGGCGACAAG	GCCTCAGCGATCTCCTTCATCTTC
Claudin2	TCTGCCCTGTACCCCAACTG	CCCAGGAAGACGGGCTTT
Occludin	ATGGTGAAGGTCGGAGTGAAC	CTCGCTCCTGGAAGATGGT
β-actin	TATGCTCTCCCTCACGCCATCC	GTCACGCACGATTTCCCTCTCAG

### Statistical analysis of data

All the data were statistically analyzed by Graph Pad Prism 5.0 (Graph Pad Software, Inc., La Jolla, CA, USA) and SPSS 19.0 one-way ANOVA method was used for the plotting and representation of results. Statistical significance values were set at (*P* < 0.05).

### Ethics approval

All experimental protocols were approved by the Animal Ethics Committee of Guangdong Ocean University, China, and were performed according to the ethical guidelines of the European Community guidelines.

### Statement for ARRIVE guidelines

We have read the journal’s policy and all the authors read and approve the manuscript. We declared that this study was carried out in compliance with the ARRIVE guidelines. The animal was raised in a stress free environment and handled with extra care. They were euthanized peacefully. All the protocols were followed according to the ARRIVE guidelines for handling the animal.

## Data Availability

Source data are provided with this paper. Any other supporting data are available from the corresponding author upon request.

## Abbreviations

COS: Chitosan
HS: Heat stress
TNF-α: Tumor necrosis factor alpha
IL: Interleukin
TJ: Tight junction protein
TLR4: Toll-like receptor 4
ACTH: Adrenocorticotropic hormone
CNS: Central nervous system
ADH: Anti diuretic hormone
HSP: Heat shock protein
NF-κB(p65): Nuclear transcription factors
MYD88: Myeloid differentiation primary response 88
